# Anti-EGFR V_HH_ Antibody under Thermal Stress Is Better Solubilized with a Lysine than with an Arginine SEP Tag

**DOI:** 10.3390/biom11060810

**Published:** 2021-05-29

**Authors:** Md. Golam Kibria, Akari Fukutani, Yoko Akazawa-Ogawa, Yoshihisa Hagihara, Yutaka Kuroda

**Affiliations:** 1 Department of Biotechnology and Life Science, Graduate School of Engineering, Tokyo University of Agriculture and Technology, 2-24-16 Nakamachi, Koganei-shi, Tokyo 184-8588, Japan; s188828x@st.go.tuat.ac.jp (M.G.K.); s200218x@st.go.tuat.ac.jp (A.F.); 2 Biomedical Research Institute, National Institute of Advanced Industrial Science and Technology (AIST), 1-8-31, Midorigaoka, Ikeda, Osaka 563-8577, Japan; yoko-ogawa@aist.go.jp (Y.A.-O.); hagihara-kappael@aist.go.jp (Y.H.)

**Keywords:** SEP tag, aggregation, single domain antibody, V_HH_, arginine SEP tag, lysine SEP tag, protein solubility

## Abstract

In this study, we assessed the potential of arginine and lysine solubility-enhancing peptide (SEP) tags to control the solubility of a model protein, anti-EGFR V_HH_-7D12, in a thermally denatured state at a high temperature. We produced V_HH_-7D12 antibodies attached with a C-terminal SEP tag made of either five or nine arginines or lysines (7D12-C5R, 7D12-C9R, 7D12-C5K and 7D12-C9K, respectively). The 5-arginine and 5-lysine SEP tags increased the *E. coli* expression of V_HH_-7D12 by over 80%. Biophysical and biochemical analysis confirmed the native-like secondary and tertiary structural properties and the monomeric nature of all V_HH_-7D12 variants. Moreover, all V_HH_-7D12 variants retained a full binding activity to the EGFR extracellular domain. Finally, thermal stress with 45-minute incubation at 60 and 75 °C, where V_HH_-7D12 variants are unfolded, showed that the untagged V_HH_-7D12 formed aggregates in all of the four buffers, and the supernatant protein concentration was reduced by up to 35%. 7D12-C5R and 7D12-C9R did not aggregate in Na-acetate (pH 4.7) and Tris-HCl (pH 8.5) but formed aggregates in phosphate buffer (PB, pH 7.4) and phosphate buffer saline (PBS, pH 7.4). The lysine tags (either C5K or C9K) had the strongest solubilization effect, and both 7D12-C5K and 7D12-C9K remained in the supernatant. Altogether, our results indicate that, under a thermal stress condition, the lysine SEP tags solubilization effect is more potent than that of an arginine SEP tags, and the SEP tags did not affect the structural and functional properties of the protein.

## 1. Introduction

Protein-based therapeutic drugs are one of the fasted growing classes of pharmaceutical products [1,2]. Among them, monoclonal antibodies (mAbs) and engineered antibody fragments are attractive therapeutic platforms [3]. In particular, a single-domain antibody (V_HH_) is the smallest antibody fragment, and unlike full-length mAbs, it consists of a single Ig domain-containing three complementarity determining regions (CDRs) [4]. Their minimal size (~15 kDa) provides better tumor and tissue penetration than the full-length mAbs [5], making it an attractive drug candidate.

Aggregation is a critical issue in protein chemistry and the development of therapeutic proteins [6]. Aggregated therapeutic proteins can cause an adverse immune response, resulting in anti-drug antibodies (ADAs) and decline their therapeutic efficiencies [7,8,9]. Proteins may aggregate in the natively folded state, partially unfolded state, or fully denatured state [10,11], and the aggregates may be stabilized through electrostatic or hydrophobic interactions [12,13,14]. Aggregation induced by thermal, physical, and chemical stresses are often irreversible.

In vitro protein solubility can be controlled by optimizing the buffer condition. Sugars, polyols, amino acids, or surfactants used as additives can act as aggregation inhibitors [15,16]. In particular, arginine has gained much attention since it can increase protein solubility without altering the protein structure [17,18]. Besides, several methods related to the addition of arginine have been reported [19,20], but none of these techniques can be used in vivo, and even in vitro, the high concentration of arginine (up to 1.0 M) makes this technique costly. Moreover, in some cases, arginine has been shown to decrease protein stability, making it unfit for high-temperature usage where the protein is usually unfolded [21].

SEP tag has emerged as a reliable and versatile technique for controlling protein solubility [13,22]. In particular, we have shown their solubilization properties for bovine pancreatic trypsin inhibitor (BPTI) [23], dengue envelope protein [24], anti-epidermal growth factor receptor (EGFR)-ScFv [25], tobacco etch virus (TEV) protease [26], *Gaussia* luciferase (GLuc) [27,28] and the third domain of EGFR [29]. Here we assessed the effect of arginine and lysine tags to control protein solubility under thermal stress. We prepared four anti-EGFR V_HH_-7D12 variants tagged with 5 or 9 arginines or lysines at the C-terminus (7D12-C5R, 7D12-C9R, 7D12-C5K, and 7D12-C9K, respectively). The untagged V_HH_-7D12 formed aggregates at high temperatures and reduced the supernatant’s protein concentration by 35%. The arginine tags were effective, but some aggregates appeared after high-temperature incubation in PB and PBS. Lysine tags with either 5 or 9 residues were the best and completely suppressed aggregation over a wide range of buffer conditions, pHs, and temperatures.

## 2. Materials and Methods

### 2.1. Mutant Design and Protein Expression

**Figure 1:**
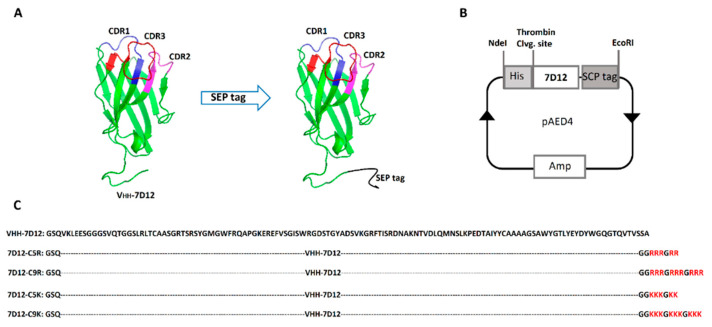
Sequence and schematic structure of the tagged V_HH_-7D12. (**A**) Ribbon structure of anti-EGFR V_HH_-7D12 and SEP tagged V_HH_-7D12 generated by Pymol [31] using the crystal structure of V_HH_-7D12 (PDB: 4KRL) [32]. The SEP tag is shown in black and was generated using Modeller. (**B**) Schematics of the anti-EGFR V_HH_ expression vector. (**C**) The amino acid sequence of V_HH_-7D12. The arginine and lysine tags were attached to the C-termini of V_HH_-7D12.

A pAED4 vector [30] was cloned with a synthetic gene that encodes anti-EGFR V_HH_-7D12 at a restriction site of NdeI and EcoR1, and the SEP tag variants were constructed by adding three repeated block of three arginines or lysines spaced by one glycine at the C-terminus of V_HH_-7D12 using site-directed mutagenesis (referred as 7D12-C9R and 7D12-C9K, respectively). Similarly, 7D12-C5R and 7D12-C5K variants were designed by adding one block of three arginines or lysines and another block containing two arginines or lysines spaced by one glycine (Figure 1). 

The V_HH_ plasmid was first transformed into the BL21 (DE3) pLysS cell line. The level of protein expression was assessed using small-scale culture (5 mL) at 37 °C. Protein expression was induced by adding 1 mM of isopropyl β-D-1-thiogalactopyranoside (IPTG) to the media at an optical density (OD) of 0.6 at 590 nm. The *E. coli* cells were collected by centrifugation 6 h after the IPTG induction, and the cells were lysed by sonication. Protein expression was analyzed by gel electrophoresis (SDS-PAGE).

### 2.2. Large Scale Protein Expression and Purification

BL21 (DE3) pLysS cell lines, transformed with V_HH_ plasmid, were grown in Luria Bertani (LB) medium (1 L) at 37 °C. Protein expression was induced with 1 mM IPTG when the OD at 590 nm of the culture reached 0.6. After 6 h of IPTG induction, the *E. coli* cells were harvested by centrifugation at 4500 rpm for 20 min at 4 °C. After sonication, V_HH_ was purified by Ni-NTA (Qiagen, Hilden, Germany), followed by ion-exchange chromatography (Gigacap-s-650 M, Tosoh Bioscience, Tokyo, Japan) according to our previously reported protocol [33]. Protein identities were confirmed by MALDI-TOF mass spectrometry (Autoflex III, Bruker Daltonics, MA, USA), and the purified proteins dissolved in Milli-Q (MQ) water was kept at −30 °C as a stock protein solution.

### 2.3. Dynamic Light Scattering (DLS)

The hydrodynamic radius (*R*_h_) of the V_HH_-7D12 variants was measured by dynamic light scattering (DLS) using a Zetasizer Nano-S system (Malvern, Worcestershire, UK). Protein samples were prepared in 20 mM Na-acetate buffer (pH 4.7) at a concentration of 0.3 mg/mL. A 100 µL polystyrene cuvette was used for DLS measurement at 25 °C. Three independent measurements were performed and averaged for the final *R*_h_ value.

### 2.4. Spectroscopic Measurements

Far-UV circular dichroism (CD) spectroscopy measurements were performed at a protein concentration of 0.15 mg/mL (10 µM) in 20 mM Na-acetate buffer (pH 4.7) using a J-820 spectropolarimeter (JASCO, Tokyo, Japan). 500 µL of the protein solution was placed in a 2 mm path-length quartz cuvette, and the spectra were collected in a continuous scanning mode from 260 to 205 nm wavelength. The spectral baseline was measured independently for each of the samples and subtracted to obtain the final spectrum. Thermal stability was measured from 15 to 90 °C using a scan rate of 1 °C/min at a wavelength of 222 nm. The midpoint temperature (*T*m) was computed by fitting the thermal denaturation curve with a two-state model without dissociation/association, using Origin 6.1.J (OriginLab Co., Northampton, MA, USA).

Sample for tryptophan fluorescence measurements was prepared according to the same protocol as for the CD measurements. Fluorescence measurements were performed on an FP-8500 spectrofluorometer (JASCO, Tokyo, Japan) using a quartz glass cuvette containing 200 µL of the sample at 25 °C. The tryptophan excitation and emission wavelength were set to 295 nm and 345 nm, respectively, and the spectra were monitored from 300 nm to 500 nm using a continuous scanning mode.

### 2.5. Surface Plasmon Resonance (SPR)

The binding affinity of the anti-EGFR V_HH_-7D12 variant was measured by surface plasmon resonance (SPR) (Biacore x100, GE Healthcare, MA, USA), as previously reported [33]. In short, the extracellular domain of human EGFR (Abcam, Cambridge, UK) was immobilized onto a CM5 sensor chip using amine coupling according to the manufacturer’s guidance. The V_HH_ protein was passed over a CM5 sensor chip at a concentration range between 0.165 and 1.25 µg/mL. All measurements were performed at a flow rate of 10 µL/min in 10 mM HBS-EP buffer pH 7.4 (10 mM HEPES, 150 mM NaCl, 3 mM EDTA, and 0.005% surfactant P20) containing 1.5 M of NaCl at 20 °C. We used 1.5 M NaCl in the buffer to avoid undesired electrostatic interaction between the negatively charged CM5 sensor chip and the positively charged tags.

### 2.6. Determination of Protein Aggregation under Thermal Stress

Protein samples were prepared at 0.5 mg/mL in four different buffers (Na-acetate buffer, pH 4.7; PB, pH 7.4; PBS, pH 7.4 and Tris-HCl buffer, pH 8.5). The protein concentration was measured based on the absorbance at 280 nm and molar extinction coefficient using a Nanodrop-2000 instrument (Thermo Fisher Scientific, MA, USA). The samples were then centrifuged at 20,000× *g* for 20 min at 4 °C, and after centrifugation, the protein concentrations were confirmed to be 0.5 mg/mL within an error of 5% arising from the removal of the debris. Protein samples were then incubated at either 60 °C or 75 °C for 45 min. Afterward, the samples were kept at room temperature for 25 min and centrifuged again at 20,000× *g* for 20 min to remove the insoluble aggregates that appeared during the heat stress. The supernatant concentration was measured for calculating the percent of the protein that formed insoluble aggregates during the incubation. The soluble aggregates remaining in the supernatant were further analyzed by measuring the Z-average (Z-ave) hydrodynamic radius (*R*_h_) using DLS. An aliquot of 100 µL of the supernatant sample was used for DLS measurement at 25 °C. Three individual measurements were performed and averaged.

## 3. Results and Discussion

### 3.1. Effect of SEP Tags on E. coli Expression of Anti-EGFR V_HH_-7D12 Variants

**Figure 2:**
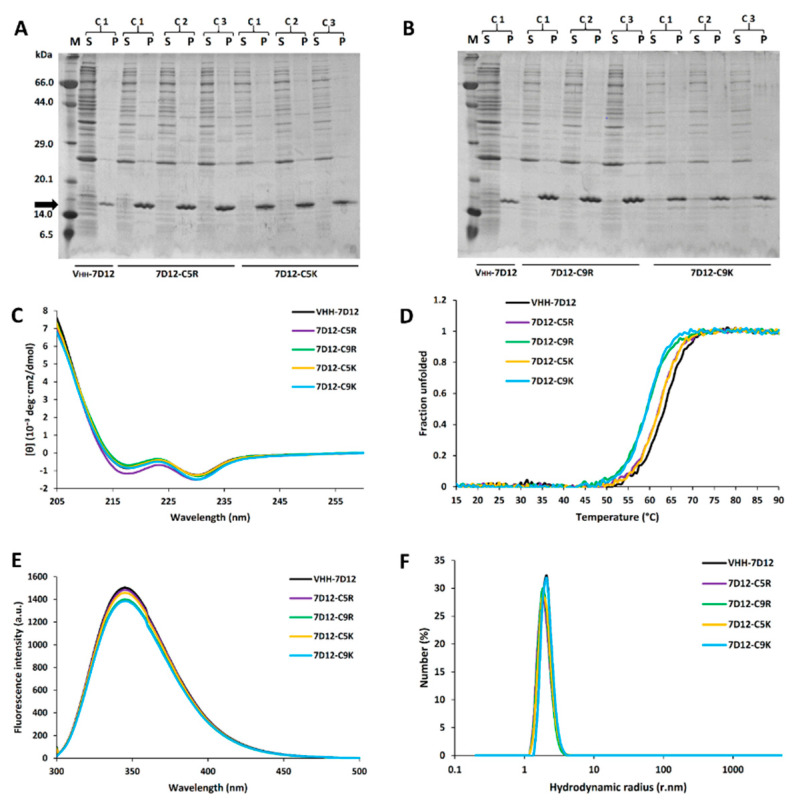
Expression and biophysical characterization of V_HH_-7D12 variants. (**A**,**B**) *E. coli* expression of V_HH_-7D12 with and without SEP tags. For each of the tagged variants, three colonies (C1~C3) were assessed. Both 5-residue arginine and lysine tags increased the V_HH_ protein expression by around 2-fold. In the 9-residue tags, only the arginine tag increased the expression. (**C**) Secondary structure content measured in 20 mM Na-acetate buffer at a protein concentration of 0.15 mg/mL (10 mM) at 25 °C. (**D**) Midpoint unfolding temperature (*T*m) measured by CD at a protein concentration of 20 mM in Na-acetate buffer (pH 4.7) at a wavelength of 222 nm. The *T*m of untagged V_HH_-7D12 was 63 °C, and by adding C5R and C5K, the *T*m decreased by 2 °C. 7D12-C9R and 7D12-C9K showed a *T*m that decreased by 4 °C. (**E**) Tryptophan fluorescence intensity in 20 mM Na-acetate buffer (pH 4.7) at 25 °C. (**F**) The number means of hydrodynamic radii (*R*_h_) measured by DLS at 25 °C.

To assess the effect of positively charged SEP tags on the *E. coli* expression of V_HH_-7D12, we first conducted small-scale culture (5 mL) at 37 °C. All of the V_HH_-7D12 variants were expressed as inclusion bodies. SDS-PAGE data showed that both C-terminal arginine tags (7D12-C5R and 7D12-C9R) increased the V_HH_ protein expression approximately twice (Figure 2A,B and Table S1), in line with our previous findings [25,26]. Likewise, the expression of 7D12-C5K increased by about two-fold, but 7D12-C9K did not show any significant expression change compared with the untagged V_HH_-7D12.

### 3.2. Biophysical and Functional Properties of V_HH_-7D12 Variants

We assessed that the tags did not affect the biophysical and biochemical nature of the V_HH_ variants. The secondary structure content of V_HH_-7D12 variants was assessed by circular dichroism (CD) using a far-UV range of 205–260 nm. The CD spectrum of the untagged V_HH_-7D12 was typical of a β-sheeted protein, and according to BestSel [34], it contained 44% of β-sheets and 1.2% of α-helices (Figure 2C and Table S2), which is in line with our previous report and the crystal structure [32,33]. The secondary structure content of the tagged variants was very similar to that of the untagged V_HH_-7D12 (Figure 2C and Table S2). 

**Figure 3:**
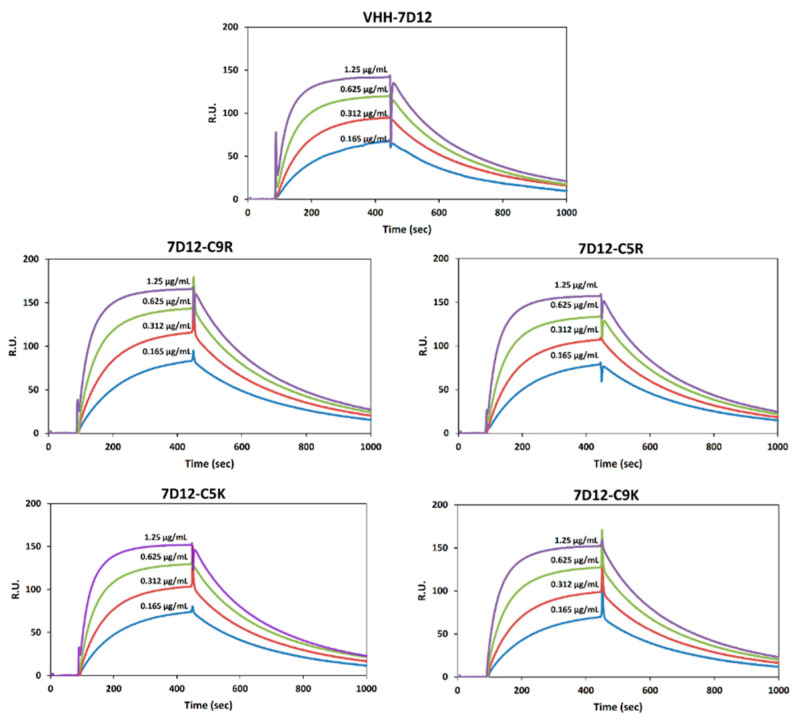
Binding activity of V_HH_-7D12 variants to EGFR using SPR: EGFR extracellular domain (20 µg/mL) was immobilized onto a CM5 sensor chip. The binding activity of tagged and untagged V_HH_-7D12 variants was analyzed at concentrations between 0.165 µg/mL and 1.25 µg/mL in HBS-EP buffer containing 1.5 M NaCl.

The tryptophan fluorescence spectra of all of the V_HH_-7D12 variants showed a maximum fluorescence intensity at 345 nm, suggesting that the tertiary structure remained unchanged (Figure 2E). Furthermore, the hydrodynamic radius (*R*_h_) measured by DLS indicated that all of the V_HH_-7D12 variants were monomeric with an *R*_h_ value of around 2 nm (Figure 2F), as expected for a small globular protein with a molecular weight of ~15 kDa. Finally, the SPR measurements indicated that all of the tagged V_HH_-7D12 variants bind to the EGFR extracellular domain, a target ligand of V_HH_-7D12, in a concentration-dependent manner, confirming their native functional properties (Figure 3 and Table 1).

### 3.3. Effect of Thermal Stress on V_HH_-7D12 Variant’s Aggregation

We first determined the midpoint temperature (*T*m) of the V_HH_-7D12 variants to fix the incubation temperature. The *T*m of the untagged V_HH_-7D12 was 63 °C, whereas the 5-residue tagged variants (7D12-C5R and 7D12-C5K) showed a 2 °C decrease (Figure 2D and Table 1), and the 9-residue tagged variants (7D12-C9R and 7D12-C9K) were reduced by 4 °C. We have no good rationale for this slight stability decrease, but we speculate that this is related to some electrostatic interaction since the decrease was correlated with the number of charged residues in the tag. In any case, the decrease was minimal and did not affect the protein’s function at ambient temperature (Table 1). For the incubation experiments, we chose 60 °C and 75 °C, where approximately half and all of the V_HH_ proteins are unfolded, respectively. The fraction of V_HH_ remaining in the supernatant before and after the heat incubation was determined in four different buffers (Na-acetate, pH 4.7; PB, pH 7.4; PBS, pH 7.4 and Tris-HCl, pH 8.5; see Materials and Methods for detailed experimental settings). Furthermore, the Z-average (Z-ave) hydrodynamic radius (*R*_h_) of the sub-visible (soluble) aggregates present in the supernatant was measured by DLS (see below).

**Figure 4:**
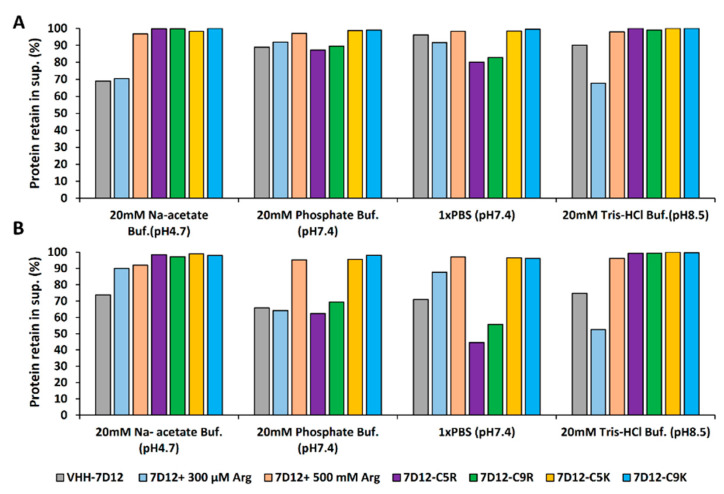
High-temperature thermal aggregation suppressive behavior of V_HH_-7D12 variants. High-temperature thermal aggregation behavior was analyzed at 60 and 75 °C, where half and all of the V_HH_s were unfolded, respectively. 0.5 mg/mL of proteins in four different buffers were heated for 45 min. After heat incubation, the samples were centrifuged at 20,000× *g* for 20 min. The amount of supernatant protein was measured just before and after heat-incubation by absorption at 280 nm to calculate the percent of the protein that formed insoluble aggregates (precipitate). Percent of protein retained in the supernatant after heat stress of tagged and untagged V_HH_-7D12 at (**A**) 60 °C and (**B**) 75 °C. Experiments at 60 °C and 75 °C were performed on the same day and using the same lot of protein.

The untagged V_HH_-7D12 formed insoluble aggregates (precipitates) in all of the four buffer conditions after a 45 min incubation at 60 °C, the supernatant’s protein concentration decreased by 4~30% depending on the buffer, with the most significant reduction occurring in Na-acetate buffer at pH 4.7 (Figure 4A and Table 2). The arginine tagged variants (7D12-C5R and 7D12-C9R) did not form precipitates in Na-acetate and Tris-HCl buffer. However, they precipitated in PB and PBS, reducing the supernatant’s protein concentration by 11~20%. Thus, under thermal stress, the arginine tags solubilization efficiency was buffer-dependent (not pH-dependent, Figure S1). In contrast, C5K and C9K tag fully inhibited the aggregation of V_HH_-7D12 in all of the buffers, including PB and PBS, and 98~100% of the V_HH_ proteins remained in the supernatant after heat stress (Figure 4A and Table 2). The difference between the effect of the arginine and the lysine tags in PB and PBS might be attributed to their side-chain properties. Namely, the guanidinium group in the arginine side-chain can form hydrogen bonds with donors in the solution (including the phosphate ions) and lead to the formation of an arginine-phosphate complex structure. This has been invoked in several studies and coined as an arginine fork [35], an arginine claw [36], or a cyclic water-phosphate-guanidinium [37]. Additionally, we assessed the effect of a C-terminal histidine tag in a control experiment since it is the only SEP tag that increases protein solubility at low pH [38]. Indeed, the histidine tag showed a strong solubilization effect at pH 4.7 (Na-acetate buffer) but not at higher pH (Figure S2), in line with our previous report [38]. As an additional control experiment, we measured the effect of free L-arginine on the protein’s solubility [15]. Arginine at a concentration of 300 µM, which corresponds to the concentration of the C9R tag, did not inhibit aggregation, but at a concentration of 500 mM, arginine had a strong solubilizing effect (Figure 4A and Table 2). 

Very similar trends were observed for V_HH_s under harsher thermal stress generated by incubation at 75 °C, where V_HH_s were essentially unfolded according to CD (Figure 4B and Table 2).

**Figure 5:**
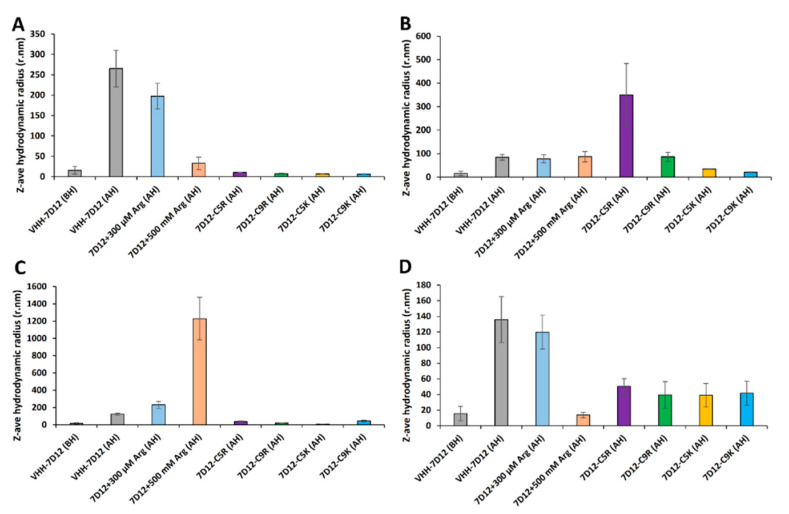
Size of sub-visible aggregates formed upon 75 °C heat incubation. The presence of sub-visible aggregates was analyzed by measuring the Z-ave hydrodynamic radius (*R*h) using dynamic light scattering (DLS). After 75 °C heat incubation, the samples were centrifuged, and 100 µL of the supernatant was used for DLS measurement at 25 °C. The *R*h of V_HH_-7D12 variants in (**A**) 20 mM Na-acetate buffer (**B**) 20 mM phosphate buffer (**C**) PBS and (**D**) 20 mM Tris-HCl.

Using DLS, we assessed the Z-ave hydrodynamic radii (*R*_h_) of the heat-induced sub-visible aggregates that remained in the supernatant after 75 °C heat incubation followed by centrifugation. In all four buffers, except in PB, the untagged V_HH_-7D12 formed aggregates with an *R*_h_ over 100 nm (Figure 5 and Table 3). The tagged V_HH_-7D12 formed some aggregates smaller than 50 nm under most conditions. The most stringent exception was PB, where the V_HH_-C5R formed aggregates of almost 400 nm (Figure 5 and Table 3). Noteworthy, the *R*_h_ of 7D12-C5K and 7D12-C9K, which essentially remained (96~99.5%) in the supernatant after 75 °C heat incubation, showed a slight increase in Na-acetate and PB (20~41 nm), but not in PBS and Tris-HCl buffer, which emphasizes the potential of the lysine tag as an aggregation inhibitor tag. L-Arginine had a similar effect on the size of the soluble aggregates formed under thermal stress, but the *R*_h_ value, particularly in PB and PBS, was larger than those of aggregates formed by the tagged variants. Note that the protein concentration was not adjusted for the amount of precipitated protein to avoid unwanted aggregation or dissociation. Overall, the lysine tag solubilized V_HH_ under heat stress, and it was monomeric, natively folded (Figures S3 and S4), and active (Figure S5 and Table 1) upon reverting the temperature to ambient temperature.

### 3.4. The SEP Tag Is a Versatile Technique for Solubilizing Proteins

Controlling protein solubility in a versatile and inexpensive way is a holy grail of protein engineering [39], especially in protein drug development. Fusion proteins such as thioredoxin [40], N utilization substance (NusA), maltose-binding protein (MBP) [41], and small ubiquitin-like modifier (SUMO) [42] have been used to solubilize proteins, but because of their large sizes, they need to be removed, which generates further cost and handling. Co-solutes can be used to control the aggregate formation, but the need of a high co-solute concentration often makes the solution hypertonic and unusable for therapeutic purposes. To date, the SEP tag is the only way for reliably controlling the protein solubility and aggregation without changing the buffer’s condition and without altering the protein’s structural and functional properties [38,43,44]. Besides, SEP tags can solubilize recombinant proteins containing multiple disulfide bonds and yield a substantial amount of fully native proteins from *E. coli* expression systems, which would otherwise not be possible [29,45].

## 4. Conclusions

In conclusion, we showed that a SEP tag made of positively charged arginine or lysine could inhibit the high-temperature thermal aggregation, where proteins are unfolded, often resulting in irreversible aggregation. Overall, the lysine tags performed somewhat better than the arginine tags, and the five residue lysine tag was the best as it increased both the expression level and solubilized V_HH_ under heat stress conditions in a monomeric, natively folded, and active state.

## Figures and Tables

**Table 1:** Midpoint unfolding temperature (*T*m) and binding affinity at 20 °C of V_HH_-7D12 variants.

Variants	MW (Da)	*T*m (°C)	SPR			
			*k*_on_ (M^−1^s^−1^)	*k*_off_ (s^−1^)	*K*_D_(M)	*K*_D_(M) ^heat^
V_HH_-7D12	13,635	63	3.2 × 10^5^ ± 5.0 × 10^4^	0.003 ± 9.7 × 10^−5^	1.1 × 10^−8^ ± 1.7 × 10^−9^	1.0 × 10^−8^ ± 1.8 × 10^−10^
7D12-C5R	14,588	61	4.1 × 10^5^ ± 5.9 × 10^4^	0.004 ± 3.8 × 10^−5^	9.7 × 10^−9^ ± 1.3 × 10^−9^	1.1 × 10^−8^ ± 5.9 × 10^−10^
7D12-C9R	15,269	61	4.5 × 10^5^ ± 6.6 × 10^4^	0.004 ±1.0 × 10^−4^	9.4 × 10^−9^ ± 1.0 × 10^−9^	1.0 × 10^−8^ ± 6.9 × 10^−10^
7D12-C5K	14,447	59	3.8 × 10^5^ ± 3.4 × 10^4^	0.003 ± 4.1 × 10^−5^	1.0 × 10^−8^ ± 1.0 × 10^−9^	1.1 × 10^−8^ ± 5.7 × 10^−11^
7D12-C9K	15,017	59	3.6 × 10^5^ ± 1.4 × 10^4^	0.004 ± 0.0001	1.2 × 10^−8^ ± 8.6 × 10^−10^	1.1 × 10^−8^ ± 7.3 × 10^−11^

^heat^: SPR measured at 20 °C after incubation at 75 °C in PBS for 45 min. The SPR data are shown in Figure S5.

**Table 2:** Amount of protein (%) retained in the supernatant after thermal stress.

Variants	60 °C				75 °C			
	Na-Acetate	PB	PBS	Tris-HCl	Na-Acetate	PB	PBS	Tris-HCl
V_HH_-7D12	68.9	89.0	96.1	90.2	73.7	65.9	70.9	74.7
7D12 + Arg *	47.3	91.8	91.6	67.8	90.1	64.2	87.7	52.6
7D12 + Arg **	96.7	97.0	98.2	97.8	92.0	95.3	97.0	96.1
7D12-C5R	99.8	87.3	80.2	100.0	98.4	62.3	44.6	99.4
7D12-C9R	99.8	89.5	82.8	98.9	97.2	69.3	55.7	100.0
7D12-C5K	98.2	98.8	98.4	100.0	99.0	95.5	96.6	99.4
7D12-C9K	100.0	98.9	99.4	100.0	98.0	98.2	96.2	99.6

* 300 µM free L-arginine ** 500 mM free L-arginine.

**Table 3:** Z-ave hydrodynamic radius (*R*_h_, nm) at 25 °C of V_HH_ variants in the supernatant after 75 °C heat incubation.

Variants	Na-Acetate	PB	PBS	Tris-HCl
V_HH_-7D12	135.7 ± 29.3	84.3 ± 12.3	122.4 ± 12.6	265.1 ± 45.0
7D12 + 300 µM Arg	119.7 ± 21.5	77.9 ± 16.7	231.6 ± 39.8	197.5 ± 31.5
7D12 + 500 mM Arg	13.6 ± 3.4	86.6 ± 21.7	1228.3 ± 246.5	32.6 ± 15.1
7D12-C5R	50.3 ± 9.8	349.9 ± 133.5	38.1 ± 5.6	10.1 ± 0.7
7D12-C9R	39.4 ± 17.0	86.2 ± 19.0	19.7 ± 1.3	7.3 ± 1.1
7D12-C5K	39.0 ± 15.0	34.5 ± 1.2	7.8 ± 0.1	6.7 ± 0.7
7D12-C9K	41.5 ± 15.2	20.5 ± 1.8	45.2 ± 7.7	6.4 ± 0.1

The Z-ave hydrodynamic radius (*R*_h_) of V_HH_-7D12 measured in 20 mM Na-acetate buffer is around 15 nm (under normal condition). The error indicates the standard deviation of three independent measurements.

## Data Availability

All data are given in the manuscript and the supplementary data.

## Supplementary Materials

The following are available online at https://www.mdpi.com/article/10.3390/biom11060810/s1, Figure S1: Effect of buffer pH on arginine tagged VHH (7D12-C5R and 7D12-C9R) solubility, Figure S2: High-temperature thermal aggregation behavior of 7D12-C6H (C-terminal six histidine), Figure S3: Secondary Structure content after 75 °C heat incubation, Figure S4: Tertiary structure properties after 75 °C heat incubation, Figure S5: Binding activity of VHH-7D12 variants to EGFR before (BH) and after (AH) heat stress using SPR; Table S1: *E. coli* expression level of VHH-7D12 variants, Table S2: Secondary structure content calculated by BestSel.
