# Supplementary material for: Anti-EGFR VHH Antibody under Thermal Stress Is Better Solubilized with a Lysine than with an Arginine SEP Tag
Source: Biomolecules. 2021 May 29;11(6):810. doi: 10.3390/biom11060810 (PMC8229009; doi:10.3390/biom11060810)
Supplement: Supplementary file 1 [file biomolecules-11-00810-s001.zip › biomolecules-1205562-supplementary.pdf]

Supporting Information

Anti-EGFR V_{HH} antibody under thermal stress is better solubilized with a lysine than with an arginine SEP tag

Md. Golam Kibria[a], Akari Fukutani[a], Yoko Akazawa-Ogawa[b], Yoshihisa Hagihara[b], Yutaka Kuroda[a *]

[a] Department of Biotechnology and Life Science, Graduate School of Engineering, Tokyo University of Agriculture and Technology, 2-24-16 Nakamachi, Koganei-shi, Tokyo, 184-8588, Japan

[b] Biomedical Research Institute, National Institute of Advanced Industrial Science and Technology (AIST), 1-8-31, Midorigaoka, Ikeda, Osaka, 563-8577, Japan

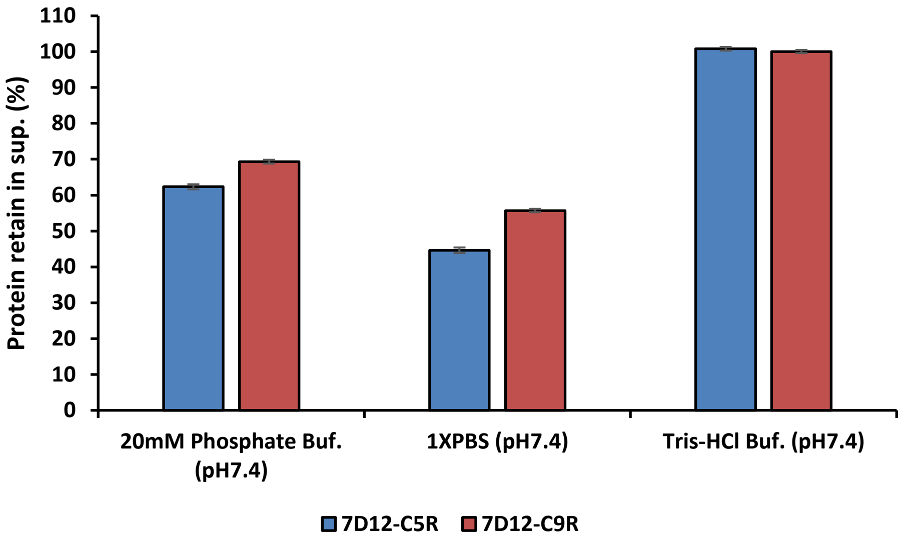

Figure S1: Effect of buffer pH on arginine tagged V_{HH} (7D12-C5R and 7D12-C9R) solubility: Protein samples were prepared at 0.5 mg/mL in three different buffers, having a pH 7.4 (phosphate (PB) buffer, phosphate buffer saline (PBS) and Tris-HCl buffer). Following the protocol mention in the main text, protein samples were incubated at 75 °C for 45 min. Percent of protein retained in the supernatant after heat stress was determined by measuring the absorbance at 280 nm. Both of the arginine tagged variant formed insoluble aggregates in PB and PBS and reduced the supernatant concentration. But they did not form any visible/insoluble aggregates in Tris-HCl buffer and retained the supernatant concentration by 100%, indicating that arginine tag solubilizing efficacy is buffer dependent not the pH. Line symbols are explained in the panel. The error indicates the standard deviation of three times concentration measurement after the heat stress using Nanodrop-2000 (Thermo Fisher Scientific, USA).

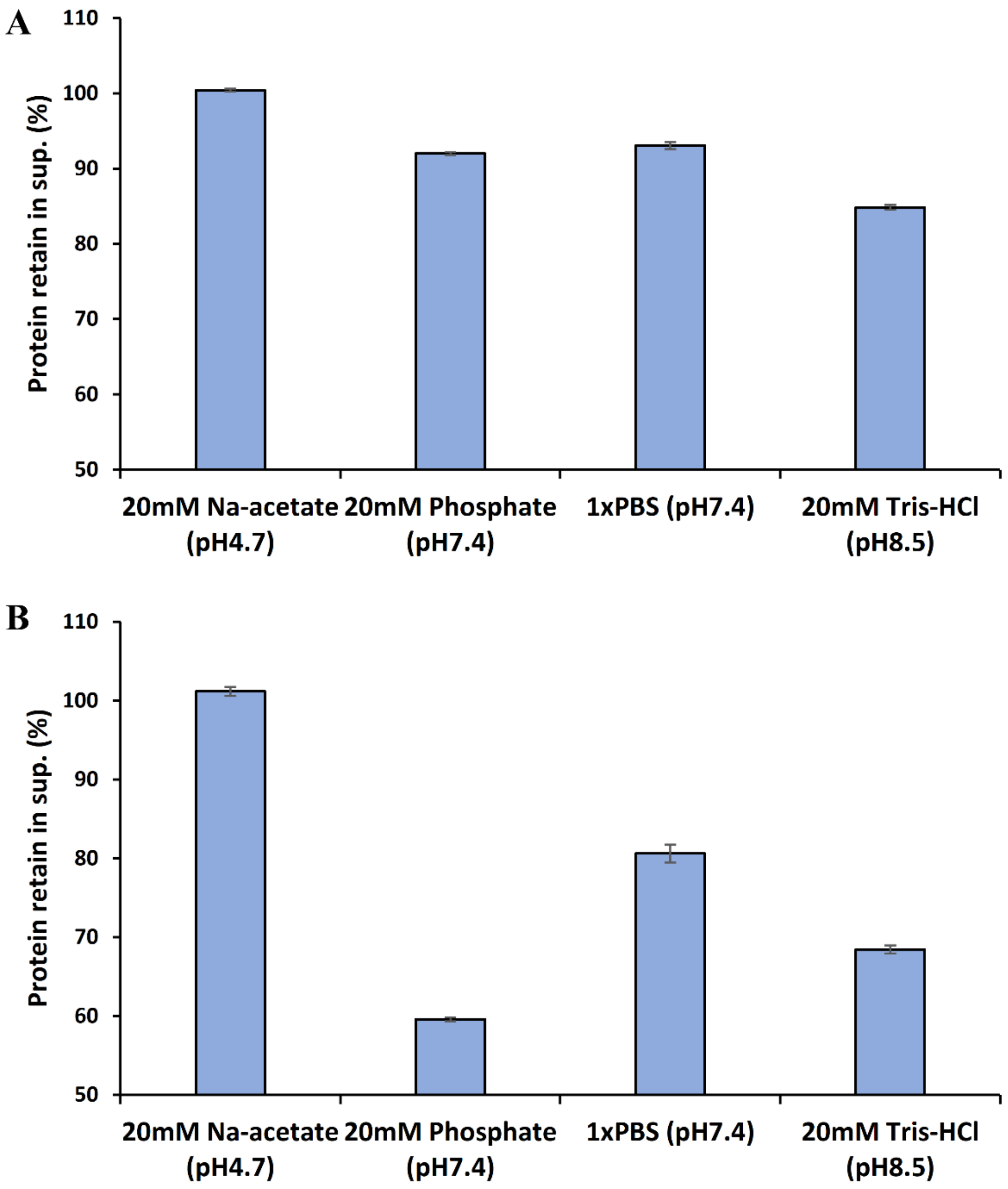

Figure S2: High-temperature thermal aggregation behavior of 7D12-C6H (C-terminal six histidine): Protein samples were prepared at 0.5 mg/mL in four different buffers (Na-acetate buffer, pH4.7; phosphate buffer (PB), pH 7.4; phosphate buffer saline (PBS), pH 7.4 and Tris-HCl buffer, pH 8.5). Following the protocol mention in the main text, protein samples were incubated at 60 °C and 75 °C for 45 min. Percent of protein retained in the supernatant after heat stress at **(A)** 60 °C and **(B)** 75 °C. Only at lower pH (Na-acetate buffer, pH 4.7) V_{HH}-C-terminal his tag showed maximum solubility and 100% protein retained in the supernatant. The error indicates the standard deviation of three times concentration measurement after the heat stress.

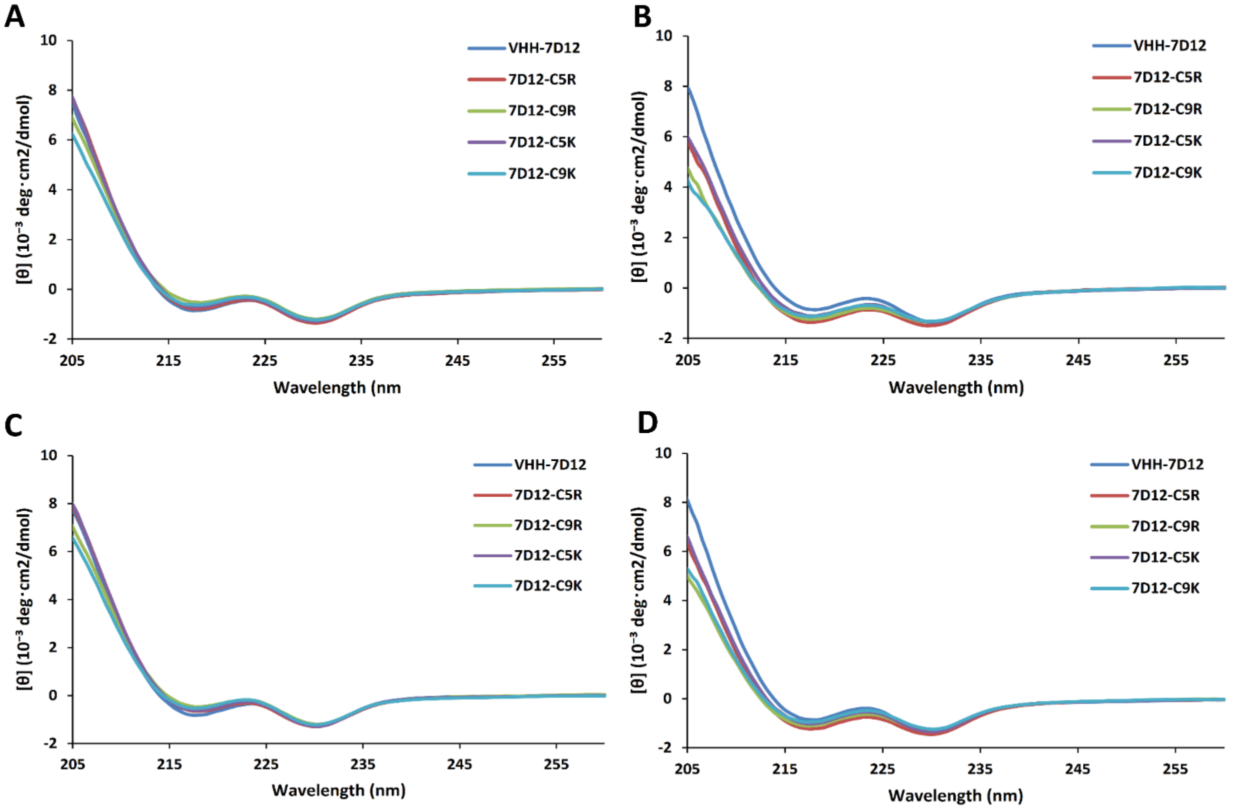

Figure S3: Secondary Structure content after 75 °C heat incubation: After incubation at 75 °C for 45 min, V_{HH} protein samples were cooled back to 25 °C and kept at this temperature for 20. Afterword, nsoluble aggregates were removed by centrifugation, and supernatant proteins have then diluted to a concentration of 0.15 mg/mL (10 µM). Far-UV circular dichroism (CD) spectra were measured in a continuous scanning mode from 260 to 205 nm wavelength (far-UV CD range) at 25 °C. CD spectra of V_{HH} protein in **(A)** 20 mM Na-acetate, pH 4.7 **(B)** 20 mM phosphate buffer, pH7.4 **(C)** 1X PBS, pH7.4 and (D) 20 mM Tris-HCl buffer, pH8.5.

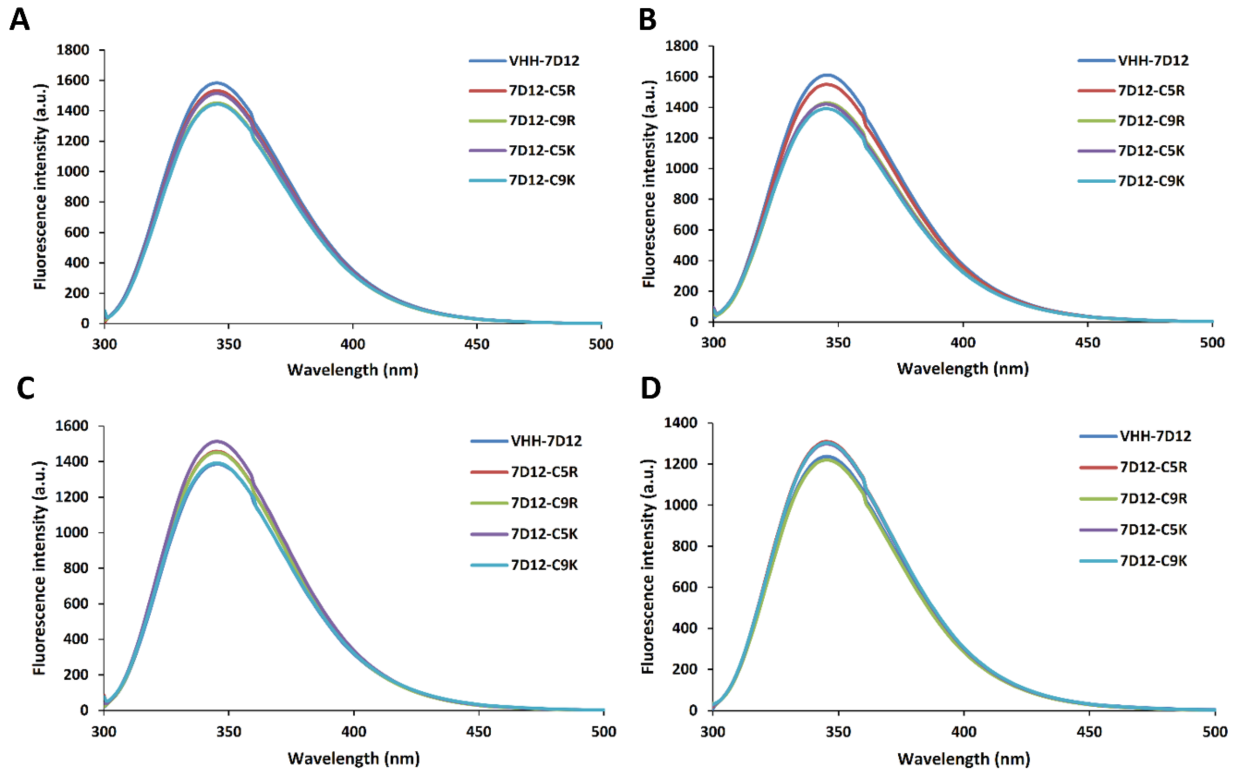

Figure S4: Tertiary structure properties after 75 °C heat incubation: After incubation at 75 °C for 45 min, the samples were cooled to 25 °C and kept at this temperature for 20. Afterword, insoluble aggregates were removed by centrifugation, and the supernatant proteins concentration were diluted to a final concentration of 0.15 mg/mL (10 µM). Tryptophan fluorescence intensity of V_{HH} protein at 25 °C in **(A)** 20 mM Na-acetate, pH 4.7 **(B)** 20 mM phosphate buffer, pH7.4 **(C)** 1X PBS, pH7.4 and (D) 20 mM Tris-HCl buffer, pH8.5.

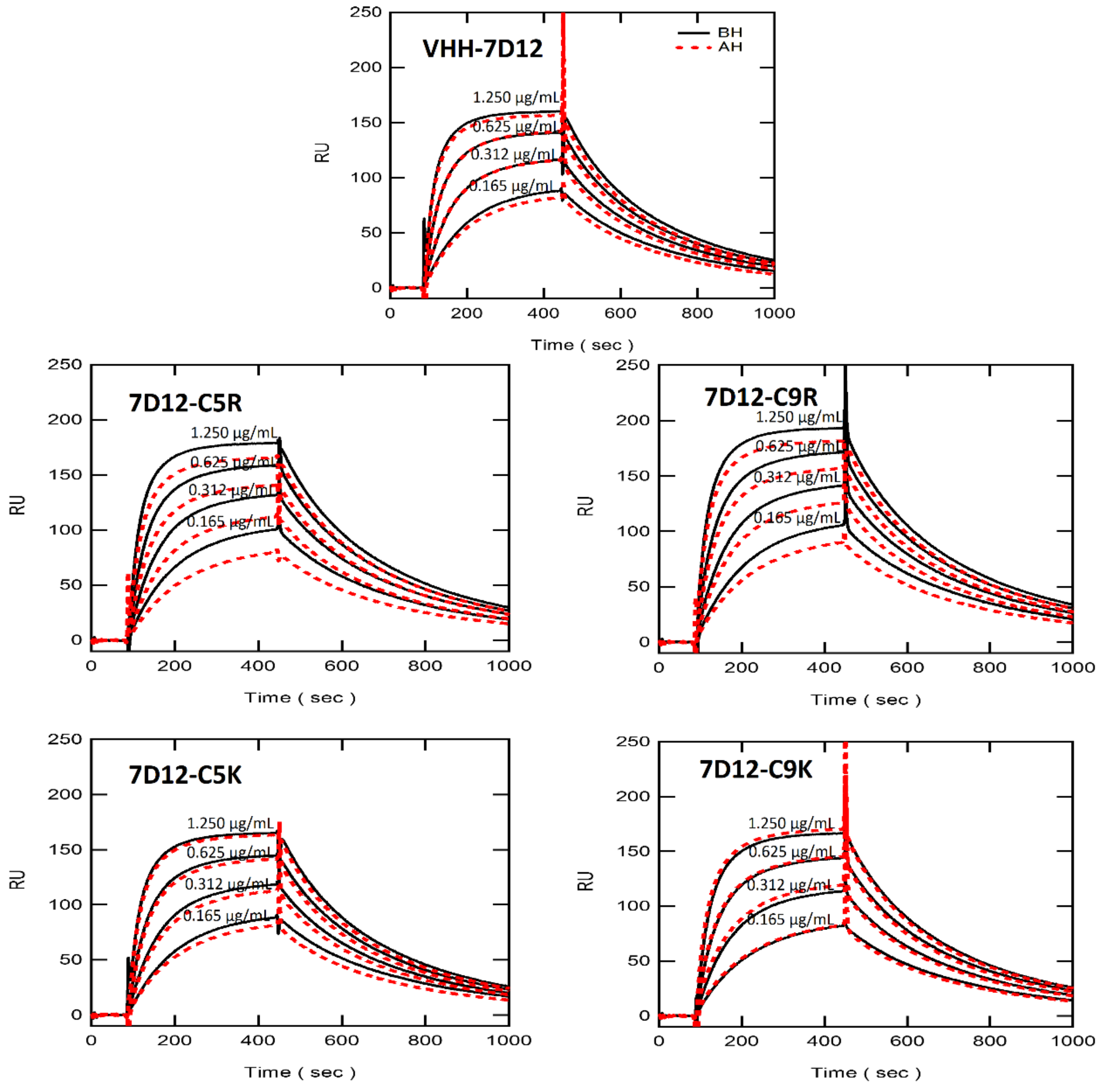

Figure S5: Binding activity of V$_{HH}$-7D12 variants to EGFR before (BH) and after (AH) heat stress using SPR: EGFR extracellular domain (20 µg/mL) was immobilized onto a CM5 sensor chip. The binding activity of tagged and untagged V$_{HH}$-7D12 variants was analyzed at concentrations between 0.165 µg/mL and 1.25 µg/mL in HBS-EP buffer containing 1.5 M NaCl. Protein samples were heated at 75 °C for 45 min in PBS, pH 7.4. Black and red dotted lines indicate the binding interaction of V$_{HH}$ protein before (BH) and after (AH) heat stress, respectively.

Table S1: *E. coli* expression level of V_{HH}-7D12 variants

	V_{HH}-7D12	7D12-C5R	7D12-C9R	7D12-C5K	7D12-C9K
Peak area	2859	5617	5647	5079	3669
Ratio	1.00	1.96	1.98	1.78	1.28

Band intensities in the SDS-PAGE electrophoresis were quantified using ImageJ software. The Peak area corresponds to the intensity of the bands.

Table S2: Secondary structural content calculated by BestSel

Sample	α-helix (%)	anti-parallel (%)	parallel (%)	turn (%)	others (%)
V_{HH}-7D12	1.2	44.1	0.0	11.1	43.6
7D12-C5R	1.6	43.9	0.0	11.1	43.4
7D12-C5K	1.6	42.8	0.0	10.8	44.8
7D12-C9R	1.7	42.1	0.0	11.4	44.8
7D12-C9K	1.4	41.4	0.0	12.2	45.0
